# Influences of dynamic load phase shifts on the energetics and biomechanics of humans

**DOI:** 10.1098/rsos.230636

**Published:** 2023-08-30

**Authors:** Qinhao Zhang, Wenbin Chen, Jiejunyi Liang, Longfei Cheng, Bo Huang, Caihua Xiong

**Affiliations:** Institute of Medical Equipment Science and Engineering, State Key Laboratory of Intelligent Manufacturing Equipment and Technology, Huazhong University of Science and Technology, Wuhan 430074, People's Republic of China

**Keywords:** load-suspended backpack, human walking, metabolic cost, dynamic load, phase shifts, trunk inclination

## Abstract

Using load-suspended backpacks to reduce vertical peak dynamic load exerted on humans can reduce metabolic costs. However, is it possible to further reduce metabolic cost by modulating dynamic load phase shift? If so, is anti-phase better than the others? In this study, we investigated the biomechanics, energetics and trunk response under phase shifts. Nine subjects wearing an active backpack with 19.4 kg loads walked on a treadmill at 5 km h^−1^ with four phase shift trials (T1–T4) and a load-locked trial (LK). Our results show that anti-phase trial (T3) assists ankle more and reduces the moment and gastrocnemius medialis activity, while T4 assists knee more and reduces the moment and rectus femoris activity. Due to the load injecting more mechanical energy into human in T3 and T4, the positive centre-of-mass work is significantly reduced. However, the gross metabolic rate is lowest in T4 and 4.43% lower than in T2, which may be because the activations of erector spinae and gluteus maximus are reduced in T4. In addition, T3 increases trunk extensor effort, which may weaken the metabolic advantage. This study provides guidance for improving assistance strategies and human–load interfaces and deepens the understanding of the energetics and biomechanics of human loaded walking.

## 1. Introduction

Carrying loads using backpacks is a common human activity. The backpack load tightened on the upper body exerts not only static loads (gravity) but also dynamic loads (accelerative force) which could reach up to 50% of the static load during walking of 5 km h^−1^ [1]. The dynamic load will increase the peak load force (static load and dynamic load) exerted on the human body, resulting in larger ground reaction forces (GRFs) [2], joint torque [3], muscle activation and fatigue [4], energy cost [3,5] and change gait [6]. In addition, carrying loads using backpacks will cause the human trunk to lean forward and more effort spent by the spine and back muscles on maintaining a stable posture [7].

To alleviate the adverse effects of carrying loads, a variety of load-carrying tools have been developed, such as load-suspended backpacks [8,9]. Load-suspended backpacks succeed in improving the walking economy by reducing the amplitude of dynamic load (also reducing peak load force) exerted on humans in the vertical direction [8–13], and minimizing the amplitude to zero could reduce 8.02% metabolic cost [1]. Improved human–load interaction by reducing the amplitude of dynamic load is beneficial to lower limb biomechanics, and thus improve the loaded waking economy [9–11,14]. Similarly, phase shift of dynamic load, defined as the phase of the peak of vertical dynamic load relative to the peak of human vertical acceleration (similar in [15]), can also change the human–load interaction. Therefore, is it possible to modulate the phase shift to improve the economy?

Dynamic load during a step cycle can be divided into positive and negative periods, which increase/decrease load force. The phase shifts redistribute the load force versus time, like moving the peak load force during middle of double support stance (DS), which is in phase with the human vertical acceleration, to other periods of gait, such as middle of single support stance (SS), which is anti-phase with the human. Dynamic load phase shift is another approach to improve the human–load interaction which may be more efficient. Some evidence suggests that anti-phase (phase shift = *π*) may have less of a metabolic cost [15,16]. During human walking, the human centre-of-mass (CoM) moves atop a stance leg that behaves approximately like a pendulum, and the mechanical work for step-to-step transitions appears to be a major determinant of the metabolic cost of walking [17–19]. It indicates reducing load force during DS may cost less work for redirecting CoM [20]. A simulation using load-suspended backpack shows that anti-phase will minimize mechanical work [15]. However, some studies do not support the idea that anti-phase is better in energy cost. Studies have shown that the highest muscular work occurs in the early single-limb support for lifting the CoM [21] since only 60% of potential energy is provided by kinetic energy, rather than the idea that a little energy is required during single-limb support according to inverted pendulum model [17]. This suggests that choosing a specific phase shift, which could reduce load force during CoM raising, may be a potential approach. Another study demonstrates that tuning phase shift could reduce force during push-off [22]. Interestingly, the nodding behaviour that evolved naturally in horses chose the anti-phase, while the nodding behaviour of other quadruped mammals chose other phase shifts [23]. Therefore, previous studies are insufficient to prove that anti-phase is the lowest energy choice for human loaded walking, and there is a lack of biomechanical and physiological results for explaining the effects of phase shifts [15,22,24]. In addition, previous studies have also shown that carrying loads using backpacks causes the trunk to lean forward and alters muscle activity [25–27]. But, most studies of load-suspended backpacks do not consider the effect of phase shifts on the trunk. For these reasons, this paper aims to investigate the trunk response, biomechanics and energetic influence of dynamic load phase shift, and confirm whether the anti-phase is the lowest metabolic cost choice.

An active backpack is required to modulate the phase shift and amplitude independently. Some studies of passive or semi-active load-suspended backpacks have analysed the possibility of modulating the amplitude and phase of the dynamic load by adjusting the stiffness or damping [14,22,28–31], but this approach cannot decouple amplitude and phase shifts of the dynamic load. In addition, the actual performance of adjusting the damping in real time is not as good as in simulation, due to imprecise parameters and gait recognition [13]. Some active backpacks have been proposed [1,24] since they can arbitrarily adjust the load motion, theoretically. Comparing with controlling load displacement on the backpack [24], controlling load vertical acceleration is more appropriate since it directly controls the dynamic load profiles [1].

In this paper, we proposed the approach of dynamic load phase shift modulation using an active backpack. To investigate the trunk response, energetics and biomechanics under dynamic load phase shifts, we measured and statistically evaluated GRFs, kinetics and kinematics, muscle activation, CoM work and gross metabolic rate in nine healthy male subjects. We hypothesize that reducing load force during double support stance (T3) and CoM raising (T4) may improve the economy. Our results show that anti-phase (T3) and T4 could assist ankle and knee, respectively. The CoM works are reduced in T3 and T4 since more mechanical energy is injected into human body from the load. But, anti-phase may lead to more trunk extensor effort even than when load is locked, which weakens the metabolic advantage and causes the lowest metabolic rate in T4. This study inspires us that strategies assisting in carrying load especially the anti-phase need to consider the negative impact on the trunk, which provides guidance for us to improve the design of the human–load interface and assistance strategy, and also give a deeper understanding of the energetics and biomechanics of human loaded walking.

## 2. Methods

### 2.1. Active load-suspended backpack with load acceleration profile tracking

An active load-suspended backpack used for this study was previously presented with the strategy of minimizing the dynamic load [1]. This backpack consisted of a passive system to suspend the payload and an active system to adjust the load acceleration. All passive and active components were integrated on a backpack frame, which connects to the wearer by the physical interface (shoulder straps and waist belt). The controllable active driving force is generated by a motor (100 W), while the passive driving force is generated by two elastic ropes. Two IMUs are installed at the waist belt and the load plate to measure the vertical acceleration of the human and the load. A force sensing resistor (FSR) is mounted on the right heel to detect the event of heel strike and calculate the gait cycle duration (*T*_GC_) in real time. The desired load acceleration is generated by measured human acceleration in real time, and the disturbance observer (DOB) and the feedforward controller are used to track the load acceleration [1].

### 2.2. Approach of phase shift modulation

**Figure 1.**
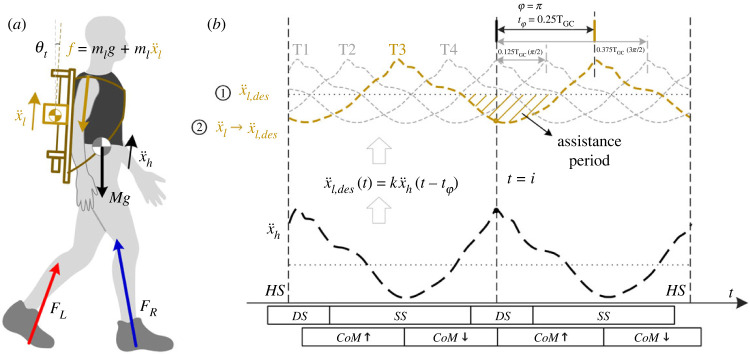
(*a*) Forces are exerted on humans using an active backpack. Load force consists of load gravity and load accelerative force (dynamic load). (*b*) The two key steps to modulate the phase shift are: ① generating desired profiles by adjusting the time delay *t*_φ_, ② tracking the desired load acceleration profiles. The 4 desired load acceleration profiles of phase shift trials at the top of the figure (grey and yellow dotted line) are generated by the measured human vertical acceleration (black dotted line) with the adjustable time delay *t*_φ_. The assistance period of T3 is marked as yellow shadow. The gait periods (DS, SS, CoM raising and CoM descending) focused on in this paper are plotted under time axes.

Human beings naturally select the pendulum-like bipedal walking gait with the CoM vertical fluctuation [32] and vertical acceleration fluctuation. Vertical acceleration profiles of load and human are similar using backpacks, while highest during the collision (about 5% gait cycle (GC)) and lowest during mid-stance (about 30% GC). The load force exerted on the human can be approximated by load gravity and dynamic load (shown in figure 1*a*). In this paper, we use dynamic load to describe the changes of load force:

2.1 $$F_{dl}(t)=m_{l}\ddot{x_{l}}(t),$$

where *m_l_* and $\ddot{x_{l}}$ are the load mass and load vertical acceleration. The negative period of dynamic load is defined as assistance period (shown in figure 1*b*) since the load force is reduced, while the positive period increases the load force. The assistance period can be moved along time by phase shift modulation to reduce external force during different gait periods or events.

The first step of phase shift modulation is to generate the desired acceleration profiles with phase shifts in real time. Desired acceleration can be expressed as

2.2 $${\ddot{x}}_{l,des}(t)=k{\ddot{x}}_{h}(t-t_{\varphi }),$$

where *k* is amplitude coefficient and *t*_φ_ is time delay of phase shift. The relationship between time delay and phase shift is: $\varphi =t_{\varphi }/0.5T_{GC}\cdot 2\pi$. In the digital controller, it can also be expressed as

2.3 $${\ddot{x}}_{l,des}(i)=k{\ddot{x}}_{h}(i-p),$$

where *i* is the current sample and *p* is the adjustable delay points. Sampled human acceleration profiles of last half gait cycle are recorded in memory (from ${\ddot{x}}_{h}(i-0.5n)$ to ${\ddot{x}}_{h}(i)$), where *n* is the sample points of each gait cycle. The second step is to track the desired load acceleration, and the control model and controller have been elucidated in previous studies [1].

In this paper, amplitude *k* is set as 0.5, because the range of load displacement on the backpack is restricted by screw length. The phase shifts are set as 0, *π*/2, *π* and 3*π*/2, with the corresponding time delay of 0*T*_GC_ (T1), 0.125*T*_GC_ (T2), 0.25*T*_GC_ (T3) and 0.375*T*_GC_ (T4). When *k* = 1, φ = 0*T*_GC_, the load acceleration is the same with human, which can be seen as load locked or ordinary backpack. When *k* = 0, phase shifts do not change the dynamic load profiles and the dynamic load is minimized [1].

According to the assistance period, the 4 phase shift trials can be divided into two groups (T1 versus T3, T2 versus T4), and their assistance periods are opposite within the group. In T3 and T1, the load force is reduced during double support stance and mid-stance, respectively. In T4 and T2, the load force is reduced during CoM raising and CoM descending, respectively.

### 2.3. Experimental protocol

**Figure 2.**
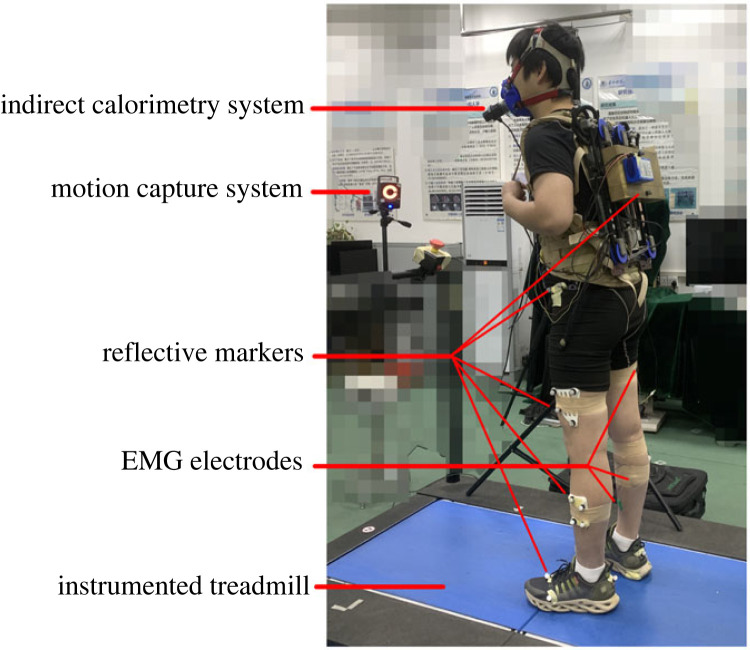
Experiment setup.

Nine healthy adults (six males, three females) participated in the experiment (age 25.56 ± 2.65 years, stature 1.72 ± 0.06 m, and weight 71.24 ± 8.63 kg (mean ± s.d.)). All participants provided informed consent before their participation, and the possible consequences of the studies were explained. The experimental protocol was approved by the Chinese Ethics Committee of Registering Clinical Trials on the use of humans as experimental subjects. The experimental protocol included two sessions: a habituation session and a testing session. Each session was performed on a separate day to avoid fatigue effects. The experiment consisted of five trials: four phase shift trials (T1–T4) and a load-locked trial (LK) by a locking mechanism on the backpack frame. In the habituation session, the subjects walk for 30 min (6 min × 5 trials) to adapt different dynamic load patterns and ensure the correct use of the active backpack. In the testing session, subjects walk for 6 min at a speed of 5 km h^−1^ with the backpack on a treadmill in five trials. All walking trials are randomly assigned orders to minimize the ordering effect, and a 15 min break was given between trials. The experiment setup is shown in figure 2.

### 2.4. Measurement and data processing

Surface electromyography (EMG) signals from 8 muscles were measured by an electromyography system (SX230, Biometrics, UK), and sampled at 1 kHz. Measured muscles include soleus (SOL), tibialis anterior (TA), gastrocnemius medialis (GAS), vastus medialis (VM), rectus femoris (RF), biceps femoris (BF), gluteus maximus (GM), erector spinae (ES). All EMGs were post-processed using custom MATLAB code.

An instrumented treadmill with force plates (AMTI, Watertown, MA, USA, 1000 Hz) was used to measure the GRFs of each leg during the experiment. The peaks and valleys of vertical GRFs were calculated for each trial.

Kinematic data were measured by a motion capture system (Vicon, Oxford, UK, 100 Hz). Retroreflective markers were attached to the lower limbs of subjects and the active backpack to record their motions. Specifically, markers were placed on the load (two), backpack (two), pelvis (four) and on the thigh (four), knee (two, only for the segment definitions), shank (four), ankle (two, only for the segment definitions), calcaneus (one), forefoot (three, distal heads of the first, second and fifth metatarsals) for each leg.

The estimated trajectory of the human CoM was calculated from the body segment positions [33]. The instantaneous CoM power is defined as dot product of CoM velocity with ground reaction force from one leg. Four periods of CoM power, defined from positive and negative intervals [19,34], collision (CO), rebound (RB), pre-load (PL) and push-off (PO), were examined. Mechanical work of CO, RB, PL, PO and sum work were calculated.

The work performed on human CoM from load force (termed as W_load_), defined as load force dot product CoM velocity, was calculated for analysing the energy flow between load and human.

Inverse dynamics analysis was performed using standard software (Visual3D, C-Motion, Germantown, MD, USA) for calculating joint angles, moments and powers for the ankle, knee and hip. Joint work was computed from joint powers.

A portable indirect calorimetry system (Oxycon Mobile, CareFusion, Germany) was used to measure the metabolic cost, and only the data during the last two minutes was calculated. The metabolic rate was normalized to each participant's body mass.

### 2.5. Statistic analysis

For each trial, means and standard errors (SEM) of gross metabolic rate, mechanical work, peak muscle activities, peaks in GRFs were calculated across subjects, with standard errors indicating inter-participant variability [35,36]. The Jarque–Bera two-sided goodness-of-fit test was used to confirm the applicability of tests that assume a normal distribution. One-way repeated measures analyses of variance (ANOVA) were conducted across T1–T4 to assess differences between phase shifts. Multiple comparisons were performed using two-sided paired *t*-test with Fisher's least significant difference (LSD) test [37]. The *p*-values marked in all figures represent the results of ANOVA across T1–T4. Then, ANOVA and multiple comparisons were conducted across T1–T4 and LK, for evaluating the advantage compared with ordinary backpacks. The significance level was set to be α < 0.05 for all analyses. All statistical analysis was conducted in MATLAB (MathWorks Inc., USA).

## 3. Results

### 3.1. Modulation of dynamic load phase shifts

**Figure 3.**
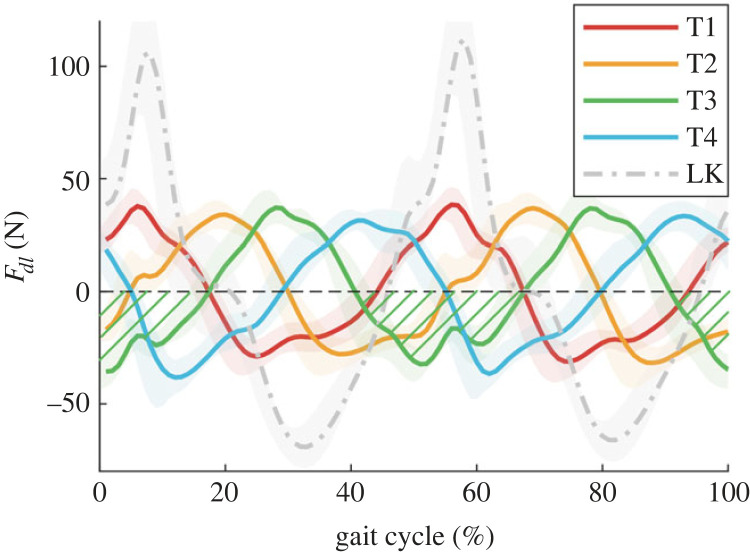
The dynamic loads in trials averaged across subjects. Green shaded areas are assistance periods in T3.

Dynamic load profiles show assistance in different gait periods under phase shift modulation (figure 3). The middle of assistance periods in T1–T4 is 31.39 ± 0.67%, 43.67 ± 0.81%, 5.06 ± 0.86%, 17.50 ± 0.74% of GC, corresponding to mid-stance, CoM descending, double support stance and CoM raising, respectively. The peak-to-peak (P-P) values of *F_dl_* are almost the same which are 78.55 ± 1.89, 74.78 ± 2.52, 80.33 ± 2.45, 79.93 ± 3.09 (N), respectively.

### 3.2. Ground reaction forces

**Figure 4.**
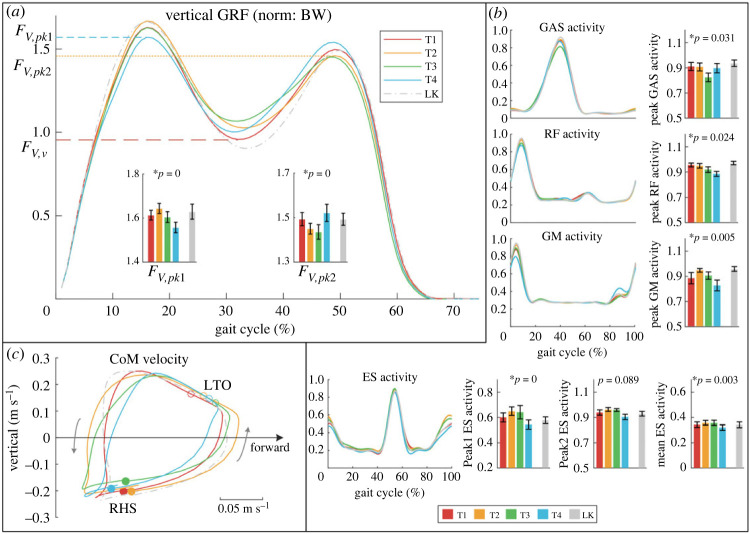
(*a*) Weight-normalized vertical GRFs averaged across subjects, and significant changes were found in peaks and valleys. (*b*) Activity of four muscles (GAS, RF, GM and ES) of 5 trials averaged across subjects and the significant differences in peak activity. Significant differences were also observed in the mean activation of ES. (*c*) The averaged CoM velocities are also termed CoM hodograph. RHS, right heel strike; LTO, left toe-off.

Significant differences are observed in the peaks and valleys of the vertical GRFs (figure 4*a*). Comparing T2 and T4, more GRF is transferred from *F_V_*_,*pk*1_ to *F_V_*_,*pk*2_ in T4 since load force is reduced during CoM raising and increased during CoM descending, while the opposite occurs in T2. Comparing T1 and T3, higher *F_V_*_,*v*_ and lower *F_V_*_,*pk*1_ and *F_V_*_,*pk*2_ are observed in T4. A complete overview of the peaks and valleys of GRFs is presented in electronic supplementary material, table S1.

### 3.3. Muscle activity

There are significant differences in peak activities for GAS, RF, GM and ES (figure 4*b*), while no significant difference was found in the other muscles. The peak activity of GAS is the lowest in T3 and reduced by 9.29% (*p* = 0.017) compared with T1. The peak activity of RF decreases with phase shift and is lowest in T4, which is 6.58% (*p* = 0.023) lower than T2. The peak activity of GM is the lowest in T4 and reduced by 12.88% (*p* < 0.01) compared with T2. Notably, there were two activity peaks of the ES muscle. The first peak demonstrates a significant difference, which is lowest in T4 and reduced by 16.5% (*p* < 0.001) compared with T2. The second peak in T4 shows a significant difference tendency (*p* = 0.089) to be the lowest. The mean activation of ES in T4 is reduced by 10.34% (*p* = 0.04) compared with T2. The specific data are presented in electronic supplementary material, table S2.

### 3.4. Joint kinematics and kinetics

**Figure 5.**
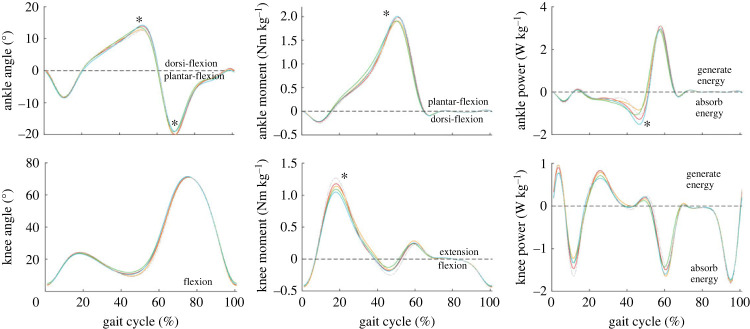
The angles, moments and powers of ankle and knee. The moment and power are normalized by human weight. *Significant differences in T1–T4.

Significantly higher peak ankle dorsi-flexion in T4 was observed than in T2 (*p* = 0.002), while there was no significant change in other joint angles (figure 5). Lower peak ankle plantar-flexion moment was observed in T2 and T3, 4.13% (*p* = 0.03) and 4.28% (*p* < 0.001) lower than T4 and T1, respectively. Lower peak knee extension moment was observed in T3 and T4, which reduced by 6.18% (*p* < 0.05) and 7.86% (*p* = 0.004) compared to T1 and T2, respectively. No significant difference was observed in hip joint angle and moment, probably because loads lead to greater effects on ankle and knee [19]. Negative ankle peak power during preload and negative ankle work was lowest in T2 and highest in T4 (*p* < 0.001, *p* = 0.023). The positive ankle peak power during push-off is higher in T4 without significant difference, while the positive ankle work is lowest in T4. Positive knee work was lowest in T3 and T4. The specific data are presented in electronic supplementary material, table S3.

### 3.5. Mechanical work and metabolic rate

**Figure 6.**
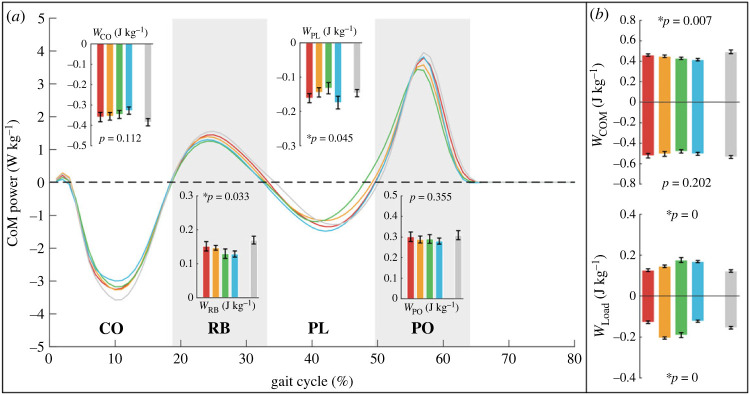
(*a*) Instantaneous CoM power from right leg. According to the positive power and negative power [19], the stance phase is divided into four periods: collision (CO), rebound (RB), preload (PL) and push-off (PO). The peak powers in the four periods show significant differences. (*b*) The CoM work and the work done by the load on the CoM (*W*_load_) that represents the energy flow between human and load.

The instantaneous CoM powers from the right leg are shown in figure 6*a*. The CoM work of CO (*W*_CO_) decreased to minimum sequentially from T1 to T4, but the difference was not significant. The CoM work of RB (*W*_RB_) was lowest in T3 and T4 and reduced by 13.41% (*p* = 0.079) and 12.52% (*p* = 0.009) compared with T1 and T2. The CoM work of PL (*W*_PL_) was highest in T4 and lowest in T3, but the positive work (*W*_PO_) did not show significant differences. Sum CoM positive work ($W_{\mathrm{CoM}}^{+}$) decreased to minimum sequentially from T1 to T4 (figure 6*b*) and was reduced by 7.02% (*p* = 0.034) in T4 compared to T2 and by 6.39% (*p* = 0.042) in T3 compared to T1. Sum negative CoM work ($W_{\mathrm{CoM}}^{-}$) was lowest in T3, but the difference was not significant.

The work performed on CoM from the load (*W*_load_) represents injecting energy into human (positive, $W_{\mathrm{load}}^{+}$) or extracting energy from human (negative, $W_{\mathrm{load}}^{-}$). $W_{\mathrm{load}}^{+}$ in T3 and T4 are higher than in T1 and T2. $W_{\mathrm{load}}^{-}$ in T2 and T3 are higher than in T4 and T1 (figure 6*b*).

**Figure 7.**
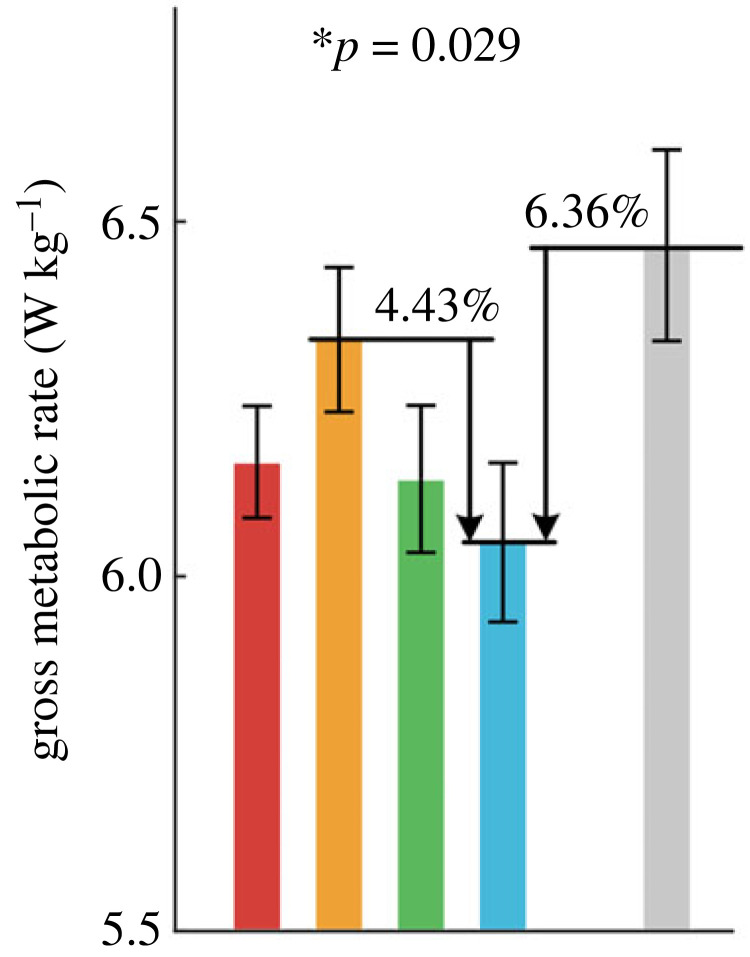
The gross metabolic rate.

The gross metabolic rate was lowest in T4 and highest in T2 (figure 7). Reducing load force during CoM raising (T4) could lead to 4.43% (*p* < 0.05) metabolic rate reduction. However, the mean metabolic rate in T3 is higher than in T4 without significant differences. Compared with LK, phase shifts reduce 6.36% (*p* < 0.01) metabolic rate at most. The specific data are presented in electronic supplementary material, table S5.

### 3.6. Trunk inclination and extension moment

**Figure 8.**
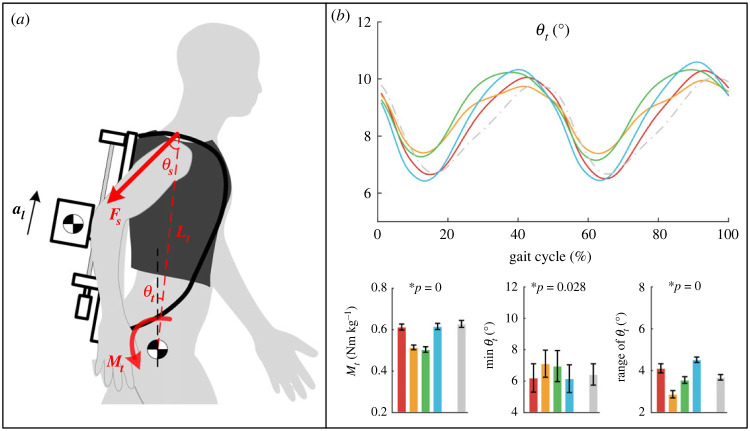
(*a*) A simple model to demonstrate the rearward moment generated by load force exerted on shoulders. (*b*) Trunk inclination angle profiles and their range and minimum angle. The mean rearward moment during the ES activation period (90%–10% GC) was also calculated.

The trunk inclination angles (*θ_t_* is shown in figure 8*a*) are calculated by markers mounted on the pelvis and neck. The range of trunk angle is greater in T1 and T4, and the minimum angle was smaller (closer to upright posture). To evaluate the assistance of the trunk extensor by rear loads, the mean rearward moment *M_t_* during the trunk extensor (ES) activation period (90%–10% GC) caused by load force exerted on shoulders was calculated. The rearward moment was higher in T1 and T4 than in T2 and T3. The specific data are presented in electronic supplementary material, table S6.

The rearward moment *M_t_* is calculated as follows and then normalized by subject weight *m_h_*. Assuming the load force is distributed proportionally between the shoulders and the waist, the proportional coefficient *μ* is 0.7 [38]. The force acting on the shoulders *F_s_* with an angle of *θ*_s_ generates a rearward moment assisting trunk extension. The rearward moment can be calculated as

3.1 $$M_{t}=\mu (m_{l}g+m_{l}a_{l})L_{t}\sin(\theta_{s})/m_{h}.$$

## 4. Discussion

How to choose a phase shift to improve walking economy is a key issue in this study. According to the muscle activity, joint moment, and CoM work, the phase shifts T3 and T4 may provide more biomechanics and energetics benefits. Reducing more load force during double-support stance (T3) assists more in the ankle push-off, which reduces the peak muscle activation of GAS, the peak ankle plantar flexion moment. In addition, *W*_RB_ is reduced in T3, which may be caused by the lower vertical CoM velocity during RB. However, the *W*_PO_ is not significantly reduced in T3, which may be due to the higher forward CoM velocity during double stance. Less load force during raising CoM (T4) mainly assists knee extension and reduces knee extension moment and peak activation of knee extensor RF. More assistance to knee joint also reduces the *W*_CO_ and *W*_RB_ since the knee joint is an important negative collision work absorber [19] and acts to lift the CoM. Although increasing the load force during CoM descending resulted in a higher peak plantar flexion moment in T4, the positive ankle work during push-off did not increase. The reason may be that the larger dorsi-flexion angle during preload helps to generate the greatest plantar flexion moment [39], which indicates the higher passive ankle plantar flexion moment and the more elastic energy through the tendon elongation [40,41].

In a stable gait, the mechanical work of the human and the load should be conserved. In T3 and T4, more mechanical work from loads is injected into human body, thereby reducing the positive CoM work. However, more negative work is extracted from human body to load in T3 than in T4, which means that more negative work performed on CoM from lower limbs was done in T4. Considering part of the negative work may come from passive biological structures such as tendons and soft tissues [34], T3 and T4 are better choices from the perspective of mechanical work.

However, the metabolic rate was lowest in T4 instead of T3 and was not as high as the CoM work showed in T1. We speculate that the different trunk response under phase shifts accounts for the metabolic cost bias. Studies have shown that moderate load on the back generates a rearward moment reducing the activation of ES [26,27]. The larger rearward moment during the activation period of ES (90%–10% GC) in T1 and T4 assists the trunk extension and reduces the activation of ES. Another similar result is GM activation. There is a high applied hip flexion moment at heel strike which needs to be counteracted by the hip extensor [42]; the larger rearward moment may lead to the reduction of GM activation in T1 and T4. These suggest that designing assistance strategies for backpack-type devices should not only consider the mechanics and energy cost of the lower limbs but also consider the impact on the trunk. To reduce or avoid unforeseen negative effects on the trunk, more load can be transferred to the waist [11] to lower the point of load force acting on the trunk to reduce the rearward or frontward moment, or, keep the load force along the direction of the trunk by symmetrical loads distributed front and rear [43] to avoid the rearward or frontward moment.

To evaluate the performance of the phase shift strategy, it is necessary to compare it with the LK. Compared with LK, the positive and negative CoM work in T1–T4 are reduced by 6.02–14.47% and 2.43–9.95%, respectively. The gross metabolic rates are reduced by 1.84–6.36% in T1–T4, which is less than the reduction of positive CoM work. The reasons may be that the CoM work cannot isolate work performed by passive structures such as tendons [44] and other soft tissues [34], contains no information on the mechanical energy changes of the body segments relative to the CoM [45,46], does not consider the counteraction of positive and negative muscle work provided by co-activated muscles [21], the cost of balance control and adaptation learning. Confusingly, the phase shift strategy did not show more metabolic reduction than minimizing dynamic load [1]. We speculate that constant load force brings more comfortable human–load interaction, which may have some potential advantages that are not directly reflected in mechanical work. Another reason is that anti-phase leads to higher ES activation even more than LK, which indicates the metabolic cost of the trunk using anti-phase strategy may be more than for ordinary backpacks. In addition, a possible factor is the empirical design of the dynamic load profiles. The profiles are generated by filtered human vertical acceleration, and the assistance period is close to 25% gait cycle duration. As mentioned above, anti-phase strategy reduces the load force during double-support stance and assists ankle push-off. However, we note that positive ankle power period during push-off is about 10–15% gait cycle duration, which is shorter than the duration of the assistance period. This inspires us that designing profiles with shorter duration and larger magnitude assistance period to match the specific interval of joint power or moment may be a better strategy.

Although the experimental results show that dynamic load phase shifts could improve the walking economy and anti-phase may lead to more trunk extensor effort, there are still a number of limitations to this study. First, the metabolic reduction of phase shifts is not more than the previous strategy [1]. We attribute this to the increased muscle effort of the trunk extensor in anti-phase. Our future work will focus on improving the human–load interface by transferring more load to the waist or symmetrically placing loads on the front and rear sides to avoid impact on the trunk. Second, the results of this paper suggest that empirical dynamic load profile design may be inefficient, and designing assistance profiles based on biological information (joint moment or power) is also our next work. In addition, the small sample size may limit the extensiveness of the conclusions in this paper, and more subject data can be collected in the future to enhance the validity of these conclusions.

## 5. Conclusion

This study investigates the influence of dynamic load phase shift strategy on trunk response, biomechanics and energetics. The anti-phase (T3) mainly assists the ankle push-off during double-support stance, and reduces GAS activation and ankle plantar flexion moment. Reducing load force during CoM raising (T4) assists knee joint more, and RF activation and knee extension moment are lowest. Due to the load injecting more mechanical energy into human body in T3 and T4, the positive CoM works are lowest. However, the gross metabolic rate is lowest in T4. This may be caused by increased trunk extensor effort in anti-phase even more than LK, which may weaken the metabolic advantage.

Although the phase shift strategy did not show plenty of metabolic advantages, our results explain the possible reason why the anti-phase strategy is not as effective as expected, which provides a basis for our next work to improve the human–load interface and assistance strategy. In addition, the mechanical energy flow of the phase shift strategy suggests that the energy injection from loads into the human may be an important consideration. This study provides a deeper understanding of the energetics and biomechanics of human loaded walking.

## Data Availability

The authors confirm that all data underlying the findings are fully available without restriction. All relevant data are presented in electronic supplementary material [47].

## References

[1] He L, Xiong CH, Zhang QH, Chen WB, Fu CL, Lee KM. 2020 A backpack minimizing the vertical acceleration of the load improves the economy of human walking. IEEE Trans. Neural Syst. Rehabil. Eng. **28**, 1994-2004. (10.1109/tnsre.2020.3011974) 32746327

[2] Birrell SA, Hooper RH, Haslam RA. 2007 The effect of military load carriage on ground reaction forces. Gait Posture **26**, 611-614. (10.1016/j.gaitpost.2006.12.008) 17337189

[3] Quesada PM, Mengelkoch LJ, Hale RC, Simon SR. 2000 Biomechanical and metabolic effects of varying backpack loading on simulated marching. Ergonomics **43**, 293-309. (10.1080/001401300184413) 10755654

[4] Hong Y, Li JX, Fong DT. 2008 Effect of prolonged walking with backpack loads on trunk muscle activity and fatigue in children. J. Electromyogr. Kinesiol. **18**, 990-996. (10.1016/j.jelekin.2007.06.013) 17720538

[5] Bastien GJ, Willems PA, Schepens B, Heglund NC. 2005 Effect of load and speed on the energetic cost of human walking. Eur. J. Appl. Physiol. **94**, 76-83. (10.1007/s00421-004-1286-z) 15650888

[6] Kinoshita H. 1985 Effects of different loads and carrying systems on selected biomechanical parameters describing walking gait. Ergonomics. **28**, 1347-1362. (10.1080/00140138508963251) 4065090

[7] Attwells RL, Birrell SA, Hooper RH, Mansfield NJ. 2006 Influence of carrying heavy loads on soldiers' posture, movements and gait. Ergonomics. **49**, 1527-1537. (10.1080/00140130600757237) 17050392

[8] Castillo ER, Lieberman GM, Mccarty LS, Lieberman DE. 2014 Effects of pole compliance and step frequency on the biomechanics and economy of pole carrying during human walking. J. Appl. Physiol. **117**, 507-517. (10.1152/japplphysiol.00119.2014) 24994885

[9] Rome LC, Flynn L, Yoo TD. 2006 Rubber bands reduce the cost of carrying loads. Nature **444**, 1023-1024. (10.1038/4441023a) 17183310

[10] Huang L, Yang Z, Wang R, Xie L. 2020 Physiological and biomechanical effects on the human musculoskeletal system while carrying a suspended-load backpack. J. Biomech. **108**, 109894. (10.1016/j.jbiomech.2020.109894) 32636004

[11] Park JH, Stegall P, Zhang H, Agrawal S. 2017 Walking with a backpack using load distribution and dynamic load compensation reduces metabolic cost and adaptations to loads. IEEE Trans. Neural Syst. Rehabil. Eng. **25**, 1419-1430. (10.1109/TNSRE.2016.2627057) 27845667

[12] Foissac M, Millet GY, Geyssant A, Freychat P, Belli A. 2009 Characterization of the mechanical properties of backpacks and their influence on the energetics of walking. J. Biomech. **42**, 125-130. (10.1016/j.jbiomech.2008.10.012) 19062021

[13] Zhang B, Liu T, Fan W, Zhang J. 2021 Sliding mode control of the semi-active hover backpack based on the bioinspired skyhook damper model. In 2021 IEEE Int. Conf. on Robotics and Automation (ICRA), Xi'an, China*,* 0 May–5 June 2021, pp. 9389-9395. (10.1109/ICRA48506.2021.9561495)

[14] Zhang B, Liu Y, Fan W, Wang Z, Liu T. 2020 Pilot study of a hover backpack with tunable air damper for decoupling load and human. In 2020 IEEE/ASME Int. Conf. on Advanced Intelligent Mechatronics (AIM), Boston, MA, USA*,* 6–9 July 2020, pp. 1834-1839. (10.1109/AIM43001.2020.9159037)

[15] Yang L, Xu Y, Zhang J, Chen K, Fu C. 2019 Design of an elastically suspended backpack with a tunable damper. In 2019 IEEE Int. Conf. on Advanced Robotics and its Social Impacts (ARSO)*, Beijing, China,* 31 October–2 November 2019, pp. 180-185. (10.1109/ARSO46408.2019.8948763)

[16] Kuo AD. 2005 Harvesting energy by improving the economy of human walking. Science **309**, 1686-1687. (10.1126/science.1118058) 16151001

[17] Donelan JM, Kram R, Kuo AD. 2002 Mechanical work for step-to-step transitions is a major determinant of the metabolic cost of human walking. J. Exp. Biol. **205**, 3717-3727. (10.1242/jeb.205.23.3717) 12409498

[18] Soo CH, Donelan JM. 2010 Mechanics and energetics of step-to-step transitions isolated from human walking. J. Exp. Biol. **213**, 4265-4271. (10.1242/jeb.044214) 21113008

[19] Huang TW, Kuo AD. 2014 Mechanics and energetics of load carriage during human walking. J. Exp. Biol. **217**, 605-613. (10.1242/jeb.091587) 24198268 PMC3922835

[20] Adamczyk PG, Kuo AD. 2009 Redirection of center-of-mass velocity during the step-to-step transition of human walking. J. Exp. Biol. **212**, 2668-2678. (10.1242/jeb.027581) 19648412 PMC2726857

[21] Neptune RR, Zajac FE, Kautz SA. 2004 Muscle mechanical work requirements during normal walking: the energetic cost of raising the body's center-of-mass is significant. J. Biomech. **37**, 817-825. (10.1016/j.jbiomech.2003.11.001) 15111069

[22] Liu M, Qian F, Mi J, Zuo L. 2022 Dynamic interaction of energy-harvesting backpack and the human body to improve walking comfort. Mech. Syst. Sig. Process. **174**, 109101. (10.1016/j.ymssp.2022.109101)

[23] Loscher DM, Meyer F, Kracht K, Nyakatura JA. 2016 Timing of head movements is consistent with energy minimization in walking ungulates. Proc. R. Soc. B **283**, 20161908. (10.1098/rspb.2016.1908) PMC5136594 27903873

[24] Yang L, Xiong C, Hao M, Leng Y, Chen K, Fu C. 2021 Energetic response of human walking with loads using suspended backpacks. IEEE/ASME Trans. Mechatron. **27**, 2973-2984. (10.1109/TMECH.2021.3127714)

[25] Talarico MK, Haynes CA, Douglas JS, Collazo J. 2018 Spatiotemporal and kinematic changes in gait while carrying an energy harvesting assault pack system. J. Biomech. **74**, 143-149. (10.1016/j.jbiomech.2018.04.035) 29752054

[26] Muslim K, Nussbaum MA. 2016 Traditional posterior load carriage: effects of load mass and size on torso kinematics, kinetics, muscle activity and movement stability. Ergonomics. **59**, 99-111. (10.1080/00140139.2015.1053538) 25994335

[27] Bobet J, Norman RW. 1984 Effects of load placement on back muscle activity in load carriage. Eur. J. Appl. Physiol. **53**, 71-75. (10.1007/BF00964693) 6542504

[28] Ackerman J, Seipel J. 2014 A model of human walking energetics with an elastically-suspended load. J. Biomech. **47**, 1922-1927. (10.1016/j.jbiomech.2014.03.016) 24709566

[29] Hoover J, Meguid SA. 2011 Performance assessment of the suspended-load backpack. Int. J. Mech. Mater. Des. **7**, 111-121. (10.1007/s10999-011-9153-7)

[30] Xie L, Cai M. 2015 Increased energy harvesting and reduced accelerative load for backpacks via frequency tuning. Mech. Syst. Sig. Process. **58–59**, 399-415. (10.1016/j.ymssp.2015.01.012)

[31] Yang L, Zhang J, Xu Y, Chen K, Fu C. 2020 Energy performance analysis of a suspended backpack with an optimally controlled variable damper for human load carriage. Mech. Mach. Theory **146**, 103738. (10.1016/j.mechmachtheory.2019.103738)

[32] Gard SA, Miff SC, Kuo AD. 2004 Comparison of kinematic and kinetic methods for computing the vertical motion of the body center of mass during walking. Hum. Mov. Sci. **22**, 597-610. (10.1016/j.humov.2003.11.002) 15063043

[33] Winter DA. 2009 Biomechanics and motor control of human movement. Hoboken, NJ: John Wiley & Sons.

[34] Zelik KE, Kuo AD. 2010 Human walking isn't all hard work: evidence of soft tissue contributions to energy dissipation and return. J. Exp. Biol. **213**, 4257-4264. (10.1242/jeb.044297) 21113007 PMC2992466

[35] Shepertycky M, Burton S, Dickson A, Liu Y-F, Li Q. 2021 Removing energy with an exoskeleton reduces the metabolic cost of walking. Science **372**, 957-960. (10.1126/science.aba9947) 34045349

[36] Nuckols RW, Lee S, Swaminathan K, Orzel D, Howe RD, Walsh CJ. 2021 Individualization of exosuit assistance based on measured muscle dynamics during versatile walking. Sci. Rob. **6**, 1362. (10.1126/scirobotics.abj1362) PMC9052350 34757803

[37] McMorris T, Delves S, Sproule J, Lauder M, Hale B. 2005 Effect of incremental exercise on initiation and movement times in a choice response, whole body psychomotor task. Br. J. Sports Med. **39**, 537-541. (10.1136/bjsm.2004.014456) 16046339 PMC1725279

[38] Lafiandra M, Harman E. 2004 The distribution of forces between the upper and lower back during load carriage. Med. Sci. Sports Exerc. **36**, 460-467. (10.1249/01.mss.0000117113.77904.46) 15076788

[39] Landin D, Thompson M, Reid M. 2015 Knee and ankle joint angles influence the plantarflexion torque of the gastrocnemius. J. Clin. Med. Res. **7**, 602-606. (10.14740/jocmr2107w) 26124905 PMC4471746

[40] Kawakami Y, Kanehisa H, Fukunaga T. 2008 The relationship between passive ankle plantar flexion joint torque and gastrocnemius muscle and achilles tendon stiffness: implications for flexibility. J. Orthop. Sports Phys. Ther. **38**, 269-276. (10.2519/jospt.2008.2632) 18448880

[41] Whittington B, Silder A, Heiderscheit B, Thelen DG. 2008 The contribution of passive-elastic mechanisms to lower extremity joint kinetics during human walking. Gait Posture **27**, 628-634. (10.1016/j.gaitpost.2007.08.005) 17928228 PMC2505349

[42] Sartor C, Alderink G, Greenwald H, Elders L. 1999 Critical kinematic events occurring in the trunk during walking. Hum. Mov. Sci. **18**, 669-679. (10.1016/S0167-9457(99)00037-8)

[43] Motmans RREE, Tomlow S, Vissers D. 2006 Trunk muscle activity in different modes of carrying schoolbags. Ergonomics **49**, 127-138. (10.1080/00140130500435066) 16484141

[44] Alexander RM. 1991 Energy-saving mechanisms in walking and running. J. Exp. Biol. **160**, 55-69. (10.1242/jeb.160.1.55) 1960518

[45] Umberger BR. 2010 Stance and swing phase costs in human walking. J. R. Soc. Interface **7**, 1329-1340. (10.1098/rsif.2010.0084) 20356877 PMC2894890

[46] Willems PA, Cavagna GA, Heglund NC. 1995 External, internal and total work in human locomotion. J. Exp. Biol. **198**, 379-393. (10.1242/jeb.198.2.379) 7699313

[47] Zhang Q, Chen W, Liang J, Cheng L, Huang B, Xiong C. 2023 Influences of dynamic load phase shifts on the energetics and biomechanics of humans. Figshare. (10.6084/m9.figshare.c.6771600) PMC10465206 37650053

